# We Need to Measure and Address Corruption and Poor Governance in Health Systems: Comment on "We Need to Talk About Corruption in Health Systems"

**DOI:** 10.15171/ijhpm.2019.44

**Published:** 2019-08-03

**Authors:** Maureen Lewis

**Affiliations:** Aceso Global, Washington, DC, USA.

**Keywords:** Healthcare, Healthcare Corruption, Governance and Healthcare

## Abstract

Hutchinson et al offer a compelling argument for greater attention to and work in corruption in healthcare. We indeed need to talk about corruption, to understand and to grasp how to prevent and address it. This paper lays out some of the rationale for how to define the research questions, how best to address corruption – arguing that governance rather than corruption may offer a preferred starting point, and highlighting some options for measuring, analyzing and stemming corruption.

Hutchinson et al^1^ offer a compelling argument for greater attention to and work in corruption in healthcare. We indeed need to talk about corruption, to understand and to grasp how to prevent and address it. Corruption entails illegal acts that are hidden, and perpetrators benefit from that opaqueness. Hence the difficulty of capturing, measuring and addressing corruption in the health sector that Hutchinson et al rightly lament. Possibly the most insidious and rooted corruption is organized crime that deals in counterfeit drugs and substandard inputs, which undermine patient treatment. Petty crime or pilfering from facilities is at the other extreme. low- and middle-income countries (LMICs) healthcare systems suffer from abuses that can include, among others, chronic absenteeism of public sector staff that leads to grossly understaffed facilities, under-the-table payments to individual physicians, nurses and orderlies where services are meant to be free at point of care, outright theft from facilities, suspicious public purchasing at various level of the system, or empty warehouses that are meant to be a source of drugs and supplies. Patients and their families are the victims who suffer both the indignity of substandard care and negative effects of inadequate or poor care. Desperate patients and their families sell assets and borrow to pay for “free” public care to save the life of a family member, which drives them into penury.^2,3^ Thus the human and financial costs of corruption are not insignificant for patients and their families.

An additional challenge is endemic corruption, where illegal actions become commonplace and are no longer perceived by the perpetrators, and often victims, as abnormal. Purchasing of public positions,^4^ chronic absenteeism^5,6^ and consistently rigged tenders^7^ offer examples of healthcare corruption and a “business as usual” attitude that allows it to persist. As a result such practices become part of the fabric of healthcare services.

Corruption not only reduces effectiveness in healthcare, as the authors argue, but it also compromises quality. The recent Lancet Commission report on Quality of Care^8^ notes that healthcare investments that fail to ensure quality may not be worth the outlay, and the US National Academy of Sciences’ *Crossing the Global Quality Chasm*^9^ devotes a chapter to corruption as a major source of poor quality. Impacts on inequality also warrant mention as informal payments, one of the best researched areas in corruption and healthcare, lead to stark inequities in access.^10^

Corruption can take various paths. At the extreme is national level fraud. One of the best recorded examples of this was high level trading in counterfeit drugs in Nigeria in the early 2000s, when 70% of drugs in circulation were designated as counterfeit; this contributed to unnecessary deaths and the exit of major pharmaceutical companies unable to compete with illicit producers. Despite death threats and other intimidation, Dr. Dora Akunyili, then Director General of the National Agency for Food and Drug Administration and Control, with support from the Minister of Health Obasanjo, was able to reduce pharmaceutical corruption, and by her departure in 2006, counterfeit drugs comprised just 16.7% of the market.^11^

While the sources of corruption vary, three elements systematically stoke illegal acts: opportunity, disincentives for honest behavior and lack of accountability. If we restrict this discussion to pubic healthcare systems in LMICs, we have a simpler analysis that represents the bulk of the problem and can be examined across countries. Lack of accountability may be the single greatest source of corruption. The Organisation for Economic Co-operation and Development’s (OECD’s) definition of accountability is “required or expected to justify actions or decisions” offers a window into why corruption persists, and at such pervasive levels, in many healthcare systems. No one is watching, data are scarce, and no one is being prosecuted, demoted, suspended or otherwise punished for bad behavior.

Although OECD country healthcare systems are far from devoid of corruption,^12^ the ability to hold public employees accountable for their performance and for deviations from acceptable norms allow standards to be set and adhered to. Defined expectations, availability of data, concern for irregularities, systematic audits and penalties for corrupt behavior help establish a culture of accountability and a law-abiding work environment. On a World Bank study tour to England and France in the late 1990s, the team queried hospitals and clinics whether staff came to work, given that they were largely assured lifetime employment. The shock of managers and their explanation for how the system worked – expectations of performance, reprimands leading to improved performance and oversight to ensure continued compliance – made it clear that the environments in LMICs looked similar to, but operated quite differently from, those in higher-income settings where norms of behavior, expectations, oversight and accountability predominate.

The same cannot be said for many LMICs. Indeed, there is often an attitude of “gaming the system.” We see this to a much lesser degree in OECD countries, for example with up-coding of diagnosis related groups^13,14^ or the close relationships between suppliers and hospital purchasers, but these are universal irregularities that also exist in LMICs. The German government attempts to reduce gaming by periodically restructuring the rules of the game in healthcare.^15^ Further, the ability to hold all stakeholders accountable allows most OECD countries to prevent and stem corruption. At the same time, they are bolstered by cultures that do not tolerate corruption, and public systems that have the infrastructure needed to oversee, identify and largely control corruption. That does not imply that corruption does not exist – it very much does^16^ – but rather that efforts to root out and prosecute need to persist to continuously counter illegal behavior.

Hutchinson et al^1^ focus on the unique and blatant corruption experiences in LMICs. LMICs face difficulties in managing corruption due to the structure of government, and the general lack of accountability in the public sector. But the structures of healthcare delivery systems contribute as well. Publicly-provided healthcare is largely hierarchical with often centralized decision-making, which means managers of hospitals and clinics lack the authority to manage effectively or to intervene when confronted with illegal, unethical or undesirable behaviors. Similarly, lower-level staff remain victims of behaviors that undermine ethical processes and have no power to intervene or even raise issues.^4^ In such a climate, going along is one option; the other is leaving the public sector. Neither is ideal, and some staff become trapped if they face limited alternative employment options. Relatedly, legal enforcement tends to be weak, auditing inadequate or excessive (too many layers and auditors can become the problem) and prosecution rare. Given such circumstances, it is hardly surprising that corruption remains prominent in LMIC healthcare systems.

## Addressing Corruption

Addressing corruption requires careful strategy and opportunistic approaches to expose, explain, understand and fix specific problems, which is why data, research and in-depth understanding of processes are so fundamental. That is where the incentives, motivations, opportunity and behaviors can be understood and explained, and together provide a basis for addressing corruption. Convening stakeholders and identifying and understanding how, where and why corruption persists offers an ideal first step, and logically leads to prioritizing where to focus and how.

Many within the system can be victims of corruption, but are tainted nonetheless by association, despite lacking the power to influence. This is where I depart from my concurrence with the approach of Hutchinson et al,^1^ as a holistic, confrontational approach may backfire. Launching an anti-corruption drive frontally can compromise good public managers, and can conceivably endanger them. Neither covering up nor taking on dangerous colleagues offers a viable alternative: both place many in government in awkward positions. That does not mean corruption should continue to be ignored.

Given the negative impact of corruption on performance, efficiency and quality, launching an effort to raise performance and improve governance as a route to undermining corruption can provide a carrot to unwrap current practices and expose problems, those linked to both corrupt practices and to simple mismanagement.^6^ Often, the line between the two is blurred. Attacking both allows more deft and effective ways to engage and address problems that impede quality services, undermine efficiency and allow poor services to persist, and at the same time identify and address corruption.

Ukraine offers an excellent example of attacking corruption through good governance initiatives. In an effort to raise performance, the health minister has updated processes and payment arrangements in the public system; shifted purchasing to transparent, information technology-based systems; made internal processes transparent; harnessed private delivery networks; and introduced diagnosis related groups to allow oversight, budgeting and tracking of hospital performance. The decline in waste and leakage of healthcare inputs has been measurable, and the responsiveness of public providers, who are now incentivized to see patients, has improved the image of and support for the public system (U. Suprun, oral communication, March 2019).

Many Eastern European countries addressed issues of absenteeism and informal payments in the 2000s based on hard evidence in an effort to regain control of their public systems. Georgia, for example, has moved to a system based on private providers and public funding with public oversight of spending, and has seen improvement in performance, and implicitly a decline in corruption.^17^

These offer examples of health system-wide efforts to root out corruption. How these were achieved and the costs and benefits of the results deserve to be understood. Research needs to be harnessed to measure the impacts and provide a body of evidence to guide policy elsewhere. But these anecdotes offer a prime example of how to approach corruption: through indirect reforms that strengthen the health system and thereby address corruption that is resistant to direct interventions.

## How to Understand, Measure and Stem Corruption

How to effectively measure corruption plagues all sectors, and all sectors similarly struggle to cope and curtail illegal practices.^18^ Successful anti-corruption efforts from various countries and sectors suggest concrete starting points for healthcare research, and these can encompass multiple disciplines and approaches, a valuable suggestion from Hutchinson et al. First, a number of countries have stemmed corruption at the national level from Poland^19^ to Rwanda,^20^ and these have had knock-on effects on the health sector that deserve attention and analysis.

Second, progress in attacking corruption entails reliance on measures of corruption. Many indicators are indirect because we are, after all, dealing with corruption, but they are nonetheless valuable. Research should harness these opportunities to get a sense of corruption in countries, industries and government. Transparency International, Global Integrity, the World Bank, the Mo Ibrahim Foundation, among other anti-corruption organizations, are generating indicators that provide a starting point. Collecting data that can be exploited for measuring irregularities in healthcare either alone or combined with other data is a useful beginning to shed light on corruption or irregularities.

Third, data systems or systematic surveys that track performance capture the nature and extent of poor performance and corruption, and offer insights on limitations in service delivery and financing. Both research questions and research itself are supported by such data. Public expenditure reviews (https://www.cbd.int/doc/case-studies/inc/PER-Core.pdf), public expenditure tracking surveys^21^ and the World Bank’s Service Delivery Indicators (https://www.sdindicators.org/), applied in numerous African countries to identify lack of complementary inputs, absenteeism and other abuses of the system that translate into under-performance,^22^ all provide a source for research on corruption. Harnessing Public Expenditure and Financial Analysis to identify and figure out how to identify and address the shortcomings reflected in the ratings is a low-cost option for helping countries simultaneously deal with both weak governance and corruption,^23^ and offers a research opportunity as well. Finally, collating and examining existing data in healthcare provision suggest another route for research, since most countries already collect but never analyze data.

Fourth, directly generating data and information in priority areas also holds promise. For example, data gathered through focus groups with clinicians, managers and back-room support to discuss bottlenecks and problems can often identify inappropriate, illegal and inefficient practices^24,25^; that would not be possible through interviews or questionnaires. Spot checks of provider performance can help reveal restrictions that compromise processes and outcomes. These can be as simple as contorted decision-making processes or the lack of data use by clinicians.

Finally, the opportunity for experimentation in interventions aimed at prevention and identification of corrupt practices is extensive, and largely untapped. For example, few efforts have assessed transparency and the role of third parties in tracking and reporting on public performance,^24^ an underutilized process for promoting accountability. Similarly, little attention has been given to reliance on forensic audits that complement the more predictable (and easily compromised) financial audits. Assessing the value of financial oversight information has promise as there sometimes are too many audits involving too many individuals; this can foster illegal behavior as supervision is diffuse and allows too many individuals to make judgements with too little oversight. Exploiting national financial audits for clues regarding use of public funding and shortcomings of public practices can help to understand where problems are likely to arise. Even those outside the sector can provide insights that guide research or investigations. Lastly, but importantly, there has been inadequate attention to straightforward solutions to procurement. Shifting to e-procurement with transparent warehousing and web- or phone-based distribution of pharmaceuticals can translate into reduced irregularities and improved availability of inputs (U. Suprun, oral communication, March 2019).^26^ It makes sense to hire the private suppliers to manage supply chains: let pharmaceutical companies deliver drugs, and suppliers deliver consumables. This offers a hypothesis and an essential area for research.

Initiatives of the nature suggested above rely on quality research to unpack, inform and analyze the issues, and on identifying the options for preventing and attacking corrupt practices. As Hutchinson et al^1^ point out, little evidence is available to guide policy or programs in this area. Much policy is based on rumor. Their call to action is well-timed and relevant, and the points in this review suggest directions for action and investment. Together, such actions initiate a process and signal directions of change, laying the groundwork for upgrading all aspects of the system and targeting corruption in the process.

## Ethical issues

Not applicable.

## Competing interests

Author declares that she has no competing interests.

## Author’s contribution

ML is the single author of the paper.
